# *KANSL1* variation is not a major contributing factor in self-limited focal epilepsy syndromes of childhood

**DOI:** 10.1371/journal.pone.0191546

**Published:** 2018-01-19

**Authors:** Kenneth A. Myers, Amelia McGlade, Bernd A. Neubauer, Dennis Lal, Samuel F. Berkovic, Ingrid E. Scheffer, Michael S. Hildebrand

**Affiliations:** 1 Epilepsy Research Centre, Department of Medicine, University of Melbourne, Austin Health, Heidelberg, Victoria, Australia; 2 Abteilung Kinderneurologie, Sozialpädiatrie und Epileptologie, Universitäts-Kinderklinik Giessen und Marburg, Standort Giessen, Giessen, Germany; 3 Stanley Center for Psychiatric Research, Broad Institute of Harvard and M.I.T., Cambridge, Massachusetts, United States of America; 4 Analytical Translational Genetics Unit, Massachusetts General Hospital, Boston, Massachusetts, United States of America; 5 Cologne Centre for Genomics, University of Cologne, Köln, Germany; 6 Department of Paediatrics, University of Melbourne, Royal Children’s Hospital, Parkville, Victoria, Australia; 7 The Florey Institute of Neuroscience and Mental Health, Heidelberg, Victoria, Australia; Radboud Universiteit, NETHERLANDS

## Abstract

**Background:**

*KANSL1* haploinsufficiency causes Koolen-de Vries syndrome (KdVS), characterized by dysmorphic features and intellectual disability; amiable personality, congenital malformations and seizures also commonly occur. The epilepsy phenotypic spectrum in KdVS is broad, but most individuals have focal seizures with some having a phenotype resembling the self-limited focal epilepsies of childhood (SFEC). We hypothesized that variants in *KANSL1* contribute to pathogenesis of SFEC.

**Materials and methods:**

We screened *KANSL1* for single nucleotide variants in 90 patients with SFEC. We then screened a cohort of 208 patients with two specific SFEC syndromes, childhood epilepsy with centrotemporal spikes (CECTS) and atypical childhood epilepsy with centrotemporal spikes (ACECTS) for *KANSL1* variants. The second cohort was also used to evaluate minor allelic variants that appeared overrepresented in the initial cohort.

**Results:**

One variant, p.Lys104Thr, was predicted damaging and appeared overrepresented in our 90-patient cohort compared to Genome Aggregation Database (gnomAD) allele frequency (0.217 to 0.116, with no homozygotes in gnomAD). However, there was no difference in p.Lys104Thr allele frequency in the follow-up CECTS/ACECTS cohort and controls. Four rare *KANSL1* variants of uncertain significance were identified in the CECTS/ACECTS cohort.

**Discussion:**

Our data do not support a major role for *KANSL1* variants in pathogenesis of SFEC.

## Introduction

*KANSL1* (OMIM 612452) encodes the KAT8 regulatory NSL complex, subunit 1 (KANSL1), a protein involved in chromatin modification [1]. Heterozygous loss-of-function mutations and deletions of *KANSL1* cause Koolen-de Vries syndrome (KdVS; OMIM 610443) which is characterized by intellectual disability and a characteristic facies. Other features include amiable personality, cardiac and renal/urologic malformations, hypotonia, skin pigmentation abnormalities, brain malformations, and seizures [2,3].

We recently analysed the epilepsy phenotypic spectrum in KdVS in 31 patients [4]. This group showed considerable heterogeneity; however, 20/31 had focal impaired awareness seizures, and all but one had onset in infancy or childhood. Seizures were often prolonged, with prominent autonomic features including vomiting and pallor. Although epilepsy was initially refractory in 18/22 patients for whom we had long term data, half of these individuals later became seizure-free [4]. Electroencephalography typically showed focal or multifocal epileptiform abnormalities, often occurring independently from both hemispheres.

The epilepsy in many patients with KdVS bore phenotypic similarities to the more common self-limited focal epilepsy of childhood (SFEC) syndromes, particularly Panayiotopoulos syndrome and childhood epilepsy with centrotemporal spikes (CECTS; also known as benign epilepsy with centrotemporal spikes and benign Rolandic epilepsy) [5]. SFEC have long been thought to occur secondary to genetic factors based on family studies [6]; however, no single gene has been identified to account for a significant fraction of cases. Although KdVS is clearly distinguished from SFEC by the presence of dysmorphic features and other abnormalities, we hypothesized that milder variants in *KANSL1* might contribute to the pathogenesis of SFEC. This study defined the frequency of *KANSL1* variants in SFEC, and evaluated whether variation in this gene is increased in SFEC compared to healthy controls.

## Materials and methods

We screened for single nucleotide variants (SNVs) in *KANSL1* in a cohort of 90 patients with childhood-onset focal epilepsy from the Epilepsy Genetics Research database. The cohort was comprised of 52 patients with CECTS, 27 with benign occipital epilepsy (BOE; Panayiotopoulos or Gastaut syndromes), 9 with atypical childhood epilepsy with centrotemporal spikes (ACECTS, previously called atypical benign focal epilepsy of childhood) and two with childhood-onset focal seizures that did not clearly fit within a syndromic diagnosis [5]. Diagnosis was based on clinical interview using a validated seizure questionnaire [7], and review of medical records; epilepsy syndromes were classified based on the International League Against Epilepsy epilepsydiagnosis.org website definitions [8]. This cohort was primarily composed of Australian or New Zealand residents, most of whom were of European descent; however, 14 were Israeli. This study was approved by the Austin Health Human Research Ethics Committee (Project H2007/02961); informed written consent was obtained from all participants or their parent or legal guardian in the case of minors or those otherwise lacking capacity to provide consent.

For SNV screening, DNA was extracted from whole blood (QIAamp DNA Blood Maxi Kit; Qiagen, Valencia, CA, USA) or saliva (Oragene, prepIT-L2P kit; DNA Genotek Inc, Ottawa, ON, Canada). Coding regions of *KANSL1*, including splice sites, were then amplified using gene specific primers (available on request) by standard protocol on a Veriti Thermal Cycler (Applied Biosystems, Carlsbad, CA). Bidirectional sequencing of all exons and flanking regions including splice sites was completed with a BigDye v3.1 Terminator Cycle Sequencing Kit (Applied Biosystems), according to the manufacturer’s instructions with sequencing products then resolved on a 3730xl DNA analyzer (Applied Biosystems). All sequencing chromatograms were compared to published cDNA sequences; nucleotide changes were detected using Codon Code Aligner (CodonCode Corporation, Dedham, MA).

We analyzed the data for likely pathogenic SNVs based on absence in the Genome Aggregation Database (gnomAD) [9]. We then studied common SNVs, comparing to allele frequencies in gnomAD in order to identify any that were overrepresented, which might contribute to pathogenicity. We evaluated the possible pathogenicity of all missense variants based on three *in silico* tools: Polymorphism Phenotyping v2 (Polyphen-2), Mutation Taster, and Grantham score [10–12].

We then analyzed whole exome sequencing data for pathogenic *KANSL1* variants in a 208-patient cohort of European individuals with CECTS or ACECTS, and 567 genetically matched controls [13]. This database was also used to assess any minor allelic variants that were overrepresented in our initial cohort.

We compared the frequency of rare variants (allele frequency < 0.001 in gnomAD) in our SFEC cohorts with the occurrence of rare variants in gnomAD using Fisher’s exact test. The sample size calculation of 180 alleles for the initial cohort is based on setting α = 0.05, using the known frequency of rare variants in gnomAD, and considering an overrepresentation of 100% to be the minimum to be considered significant.

## Results

From the initial 90-patient cohort, no rare *KANSL1* variants were found. We identified 23 minor allelic variants, some of which were overrepresented in our cohort compared to gnomAD (Table 1). Of these, 16 were unlikely to be significant based on being synonymous (9) or intronic (7). The remaining seven variants were all missense and *in silico* testing suggested that only one was likely to be damaging, c.311A>C (p.Lys104Thr).

**Table 1:** Missense variants identified in screen of 90 patients with SFEC (NM_015443.3).

Variant	SNP Identifier	AF in SFEC Cohort	AF in gnomAD (Total)	AF in gnomAD (European)	Polyphen-2	Mutation Taster	Grantham	p *
c.311A>C, p.Lys104Thr	rs17585974	0.217 (39/180)	0.116 (31856/274956)	0.172 (21546/125108)	0.999 (Probably damaging)	Disease Causing	78	1.0 x 10^−4^
c.662C>T, p.Thr221Ile	rs17662853	0.106 (19/180)	0.118 (32559/275816)	0.162 (20431/125940)	0.557 (Possibly damaging)	Polymorphism	89	0.72
c.673A>G, p.Asn225Asp	rs35643216	0.217 (39/180)	0.112 (30415/271952)	0.168 (20801/123560)	0.420 (Benign)	Polymorphism	23	4.6 x 10^−5^
c.2152T>C, p.Ser718Pro	rs34043286	0.244 (44/180)	0.143 (39507/277130)	0.212 (26792/126648)	0.000 (Benign)	Polymorphism	74	4.4 x 10^−4^
c.2294C>T, p.Ala765Val	rs151099014	0.006 (1/180)	0.000512 (141/275482)	0.000571 (72/126192)	0.012 (Benign)	Disease Causing	64	0.089
c.3029C>T, p.Pro1010Leu	rs7220988	0.289 (52/180)	0.374 (103519/276944)	0.397 (50265/126518)	0.000 (Benign)	Polymorphism	98	0.021
c.3254T>C, p.Ile1085Thr	rs34579536	0.244 (44/180)	0.142 (39084/275478)	0.211 (26471/125706)	0.000 (Benign)	Polymorphism	89	2.4 x 10^−4^

* p value is calculated based on comparing AF in SFEC cohort to AF in gnomAD with Fisher’s exact test; using Bonferroni correction, significance threshold should be p = 0.05/7 = 0.007. Note that this is an inspection of the data and not a rigorous statistical test, since we did not have pre-test hypothesis that these specific variants would be overrepresented.

Abbreviations: AF (Allele frequency); gnomAD (Genome aggregation database); SFEC (Self-limited focal epilepsy of childhood); SNP (Single nucleotide polymorphism).

Although inspection of the data using Fisher’s exact test suggested this variant was overrepresented in our cases (0.217) compared to gnomAD (0.120); this variant was found with allele frequency of 0.162 in the CECTS/ACECTS cohort, and did not differ significantly from the allele frequency in the European control group (0.167).

*KANSL1* screening in a replication cohort comprising 208 patients with CECTS/ACECTS identified four rare (allele frequency < 0.001 in gnomAD) missense variants (Table 2). Although none were predicted pathogenic by all *in silico* tools, two were predicted to be “probably damaging” by Polyphen-2 (p.Asn230Asp and p.Arg181Trp) and one “disease causing” by Mutation Taster (p.His212Arg).

**Table 2:** Rare missense variants identified in replication cohort comprising 208-patient CECTS/ACECTS cohort (NM_015443.3).

Variant	SNP Identifier	AF in CECTS/ ACECTS Cohort	AF in gnomAD (Total)	AF in gnomAD (European)	Polyphen-2	Mutation Taster	Grantham
c.541C>T, p.Arg181Trp	rs375225315	0.002 (1/416)	0.0000162 (4/247270)	0.00000860 (1/116232)	0.994 (Probably damaging)	Polymorphism	101
c.635A>G, p.His212Arg	rs141110759	0.002 (1/416)	0.000365 (101/277058)	0.000529 (67/126634)	0.220 (Benign)	Disease Causing	29
c.688A>G, p.Asn230Asp	rs34756740	0.002 (1/416)	0.000609 (150/246126)	0.00114 (127/111632)	0.979 (Probably damaging)	Polymorphism	23
c.1000A>C, p.Asn334Asp	rs112150341	0.002 (1/416))	0.000053 (13/246254)	0.000107 (12/111710)	0.219 (Benign)	Polymorphism	68

Abbreviations: ACECTS (Atypical childhood epilepsy with centrotemporal spikes); AF (Allele frequency); CECTS (Childhood epilepsy with centrotemporal spikes); gnomAD (Genome aggregation database); SNP (Single nucleotide polymorphism).

No loss-of-function variants were identified in either cohort.

In comparison to our finding of four rare *KANSL1* variants in a total cohort size of 298 individuals (596 alleles), there are 4384 rare missense *KANSL1* variants in the gnomAD database, which has good coverage data on ~240 000 alleles. Based on this, we would expect to find ~11 rare variants in our total cohort of 298 patients if the frequency was the same as in healthy individuals. Thus, we found fewer rare *KANSL1* variants in our SFEC cohort than would be expected to be present by chance based on healthy population data (p = 0.03).

## Discussion

Although the epilepsy phenotype in KdVS caused by heterozygous mutation of *KANSL1* bears some resemblance to SFEC, this study did not provide strong evidence that *KANSL1* variants contribute to the genetics of SFEC. We identified several rare missense variants predicted to be damaging by *in silico* analysis, as well as increased frequencies of some minor allelic variants; however, the biological significance of these findings is unclear.

Our finding of an apparent overrepresentation of the c.311A>C variant in the SFEC cohort was enticing as this SNV is predicted “probably damaging” and “disease causing” by Polyphen-2 and Mutation Taster, and may be an important amino acid modification site [14]. Of further interest is the observation that, despite the high frequency of heterozygote carriers in gnomAD, there are no homozygotes reported. This raised the possibility that the variant could be disease-causing via autosomal recessive inheritance. However, on reviewing the frequency of the variant in the 208-patient cohort of CECTS/ACECTS, there was no difference in frequency between affected patients and controls. This illustrates the challenges of evaluating the biological significance of genetic variants in a single, relatively small, cohort, and emphasizes the importance of using a second group of affected individuals to confirm findings.

We also identified four rare variants in the 208-patient CECTS/ACECTS cohort, two of which were predicted “probably damaging” by Polyphen-2 and a third “disease causing” by Mutation Taster. However, though these variants are potentially damaging, all were present in gnomAD at least four times, suggesting they are unlikely to be significant. Furthermore, the proportion of rare variants in our patients was not greater than the occurrence of rare variants in gnomAD, suggesting these variants may have been present by chance. In general, the gnomAD data indicates that missense variants are unlikely to be significant in *KANSL1*. This gene has a missense Z score of 0.49 and any score < 3 indicates that a gene is very tolerant of missense variation [9]. The lack of nonsense, splice site or small indel alleles within coding regions or splice sites of *KANSL1* in our patients, that would be expected to be associated with disruption of gene expression and loss-of-function, did not warrant pursuit of such variants in other non-coding regulatory regions (e.g. promoter) of the gene.

In summary, our data suggest that variants in *KANSL1* are not a significant cause of SFEC syndromes. We note that, as a chromatin-modifier, *KANSL1* has downstream effects on the transcription of hundreds of genes; a better understanding of the underlying genetics of SFEC may lie in a more thorough interrogation of the roles of these genes.
